# Fluid viscoelasticity promotes collective swimming of sperm

**DOI:** 10.1038/s41598-017-03341-4

**Published:** 2017-06-09

**Authors:** Chih-kuan Tung, Chungwei Lin, Benedict Harvey, Alyssa G. Fiore, Florencia Ardon, Mingming Wu, Susan S. Suarez

**Affiliations:** 1 Department of Biomedical Sciences, Cornell University, Ithaca, NY 14853 USA; 2 Department of Biological and Environmental Engineering, Cornell University, Ithaca, NY 14853 USA; 3 Department of Physics, North Carolina A&T State University, Greensboro, NC 27411 USA; 4 Mitsubishi Electric Research Laboratories, Boston, MA 02139 USA

## Abstract

From flocking birds to swarming insects, interactions of organisms large and small lead to the emergence of collective dynamics. Here, we report striking collective swimming of bovine sperm in dynamic clusters, enabled by the viscoelasticity of the fluid. Sperm oriented in the same direction within each cluster, and cluster size and cell-cell alignment strength increased with viscoelasticity of the fluid. In contrast, sperm swam randomly and individually in Newtonian (nonelastic) fluids of low and high viscosity. Analysis of the fluid motion surrounding individual swimming sperm indicated that sperm-fluid interaction was facilitated by the elastic component of the fluid. In humans, as well as cattle, sperm are naturally deposited at the entrance to the cervix and must swim through viscoelastic cervical mucus and other mucoid secretions to reach the site of fertilization. Collective swimming induced by elasticity may thus facilitate sperm migration and contribute to successful fertilization. We note that almost all biological fluids (e.g. mucus and blood) are viscoelastic in nature, and this finding highlights the importance of fluid elasticity in biological function.

## Introduction

In biology, collective movement spontaneously occurs in diverse systems ranging from swimming bacteria^1^, swarming insects^2^, and flocking birds^3–5^ to dancing crowds in a rock concert^6^. In all of these systems, one finds that the interactions of the constituents lead to collective group behavior, similar to phase transitions in physics, in which order emerges by either lowering random thermal fluctuations (temperature) or increasing the inter-particle coupling^7, 8^. For example, flocking birds were found to operate close to the transition point^4^. Large, complex, multicellular animals can interact with each other through cognition, while cells and single cell organisms communicate typically through chemical (e. g. chemical gradients)^9–11^ or physical (e. g. hydrodynamic and steric) cues^12–16^. Here we report a novel model system where sperm cells swim collectively in response to the viscoelasticity of medium that mimics biological fluids.

Most biological fluids are viscoelastic in nature. For example, some bacteria form biofilms in a viscoelastic polysaccharide matrix^17^. In mammals, bodily fluids such as blood^18^ and mucus^19–21^ are also viscoelastic. In human fertility, the elastic property of cervical mucus is a key indicator for determining whether a woman is in the fertile period of her hormonal cycle^22^. In humans and cattle, successful fertilization after mating requires sperm to swim through cervical mucus and other viscoelastic secretions along the female reproductive tract^23^. Recent findings have called attention to the critical role that the co-evolution of sperm and the female reproductive tract plays in facilitating sperm migration through the female tract. It has been discovered that the internal surface architecture of the female tract^24^ and fluid flows through the tract^25^ direct sperm migration^21^. Here we discovered a new aspect in which the female tract could regulate sperm migration. We found that the viscoelastic properties of biological fluids promotes sperm-sperm interaction leading to a striking pattern of collective swimming. This work highlights the importance of fluid elasticity, and notes that fluid elasticity can be a result of co-evolution of cell and its surrounding physiological environment in facilitating biological function. Our observation also provides an evolutionary pathway for the development of sperm cooperation observed in several species of rodents^26, 27^.

## Results

### Bovine sperm and model viscoelastic fluids

We used bovine sperm, *Bos taurus*, as our model swimmer. Bovine sperm provide a good model system because (1) the swimming characteristics and anatomic structures have been well described, (2) *in vitro* fertilization has been successfully established for bovine sperm, and (3) bulls inseminate sperm into the anterior vagina near the entrance to the cervix as occurs in humans but not in most other nonprimate mammalian species. Bovine sperm have a paddle-shaped head that is roughly 10 μm long, 5 μm wide, and 1 μm thick. The head is connected to a single flagellum that is 50–60 µm long and 1 μm in diameter at its connection to the sperm head, tapering to ∼200 nm in diameter at the distal end. In basic sperm medium, they swim in one of two ways: one with a rolling of the body and the other with planar beating of the flagellum^28–31^ (See Supplementary Movie 4, 6).

We used long-chain polyacrylamide (5–6 MDa, LC-PAM) dissolved in standard medium as our model viscoelastic fluid. The viscoelasticity of the LC-PAM solution was designed to mimic the physical properties of bovine cervical mucus during the fertile period of the hormonal cycle^21^. To study the impact of viscosity on sperm swimming behaviour, we used 3% polyvinlpyrrolidone (360 kDa, PVP). A comparison of the rheological properties of these fluids are shown in Supplementary Fig. 1.

As mentioned above, the most likely site for bovine sperm clustering *in vivo* is at the site where semen is deposited, in the cranial vagina at the external os of the cervix. The concentration of sperm in bovine semen ranges from 300 to 2500 million/mL^32^. Similarly, we used a highly concentrated aliquot of sperm in standard medium to seed the chamber. From the seeding hole, sperm rapidly entered the chamber filled with test solution, just as naturally inseminated sperm rapidly enter the cervical mucus from the semen^33^. Sperm also encounter viscoelastic substances in the oviduct, where concentrations are highly heterogeneous along the tract^33^.

### Sperm collective movements occurred in viscoelastic fluid, but not in viscous fluid

**Figure 1:**
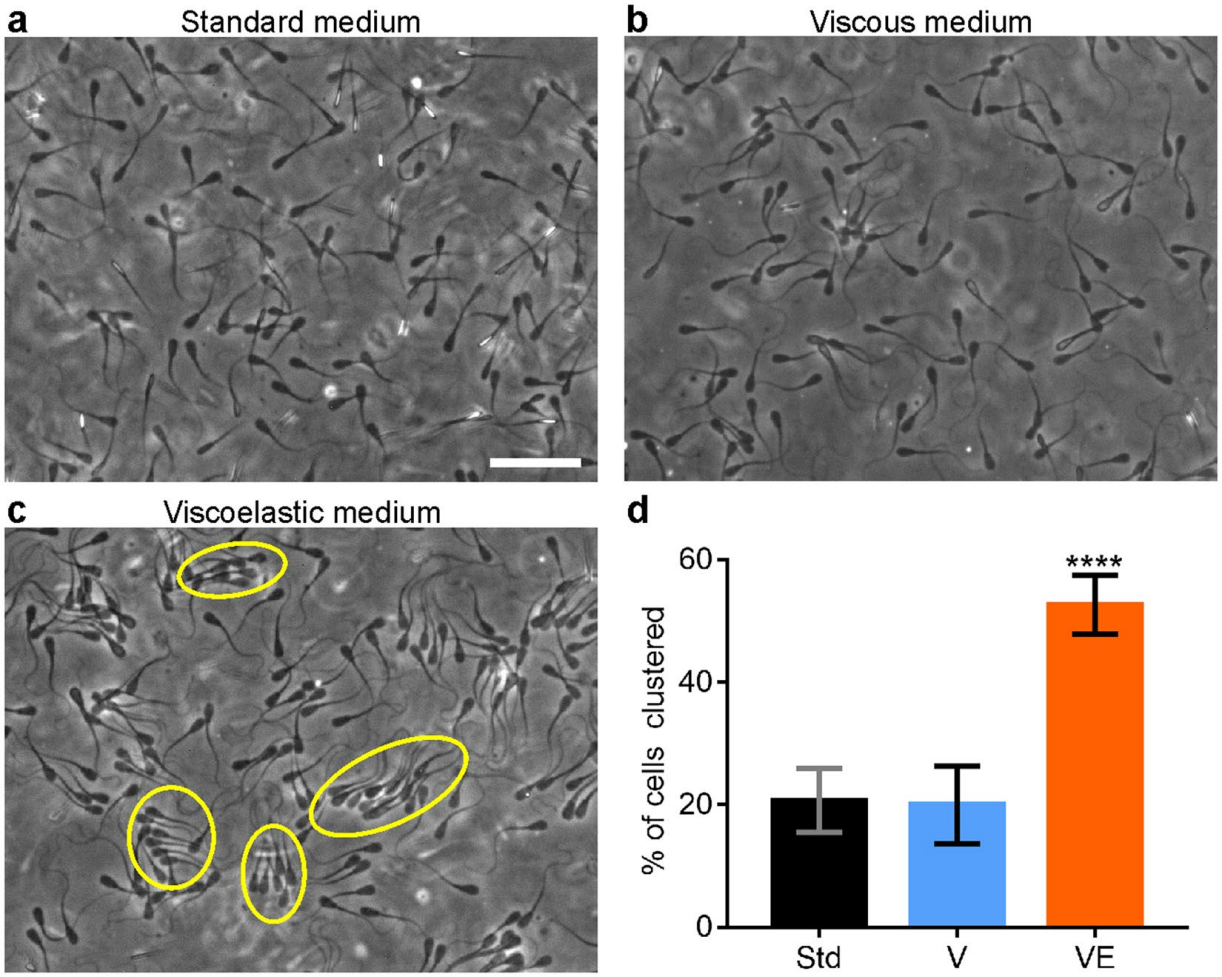
Different collective behaviours of swimming sperm in Newtonian and viscoelastic fluids. (**a**) In standard medium, sperm swam individually. Scale bar: 50 μm. (**b**) In a Newtonian viscous medium (3% PVP), most sperm swam individually. (**c**) In viscoelastic medium (1% LC-PAM), sperm swam collectively and formed clusters. (**d**) Percentage of sperm found in clusters (including pairs) in standard medium (Std), Newtonian viscous medium (V), and viscoelastic medium (VE). The definition of cluster is when the sperm are within 17.5 µm each other, and their head orientation is within 20°. ****p < 0.0001, compared to standard medium, from one-way ANOVA with multiple comparison.

We examined collective sperm swimming on a flat surface in three different types of fluids: a Newtonian fluid with low viscosity (standard medium), a Newtonian fluid with high viscosity (3% PVP in standard medium), and a Non-Newtonian viscoelastic fluid (0.4–1% LC-PAM in standard medium). In order to avoid unwanted fluid flows, we used a microfluidic chamber we had developed previously^31^, where the viewing chamber is 2.47 mm wide, 2 cm long, and 120 µm deep. In all fluids, most sperm swam near a solid surface due to hydrodynamic and other interactions with the surface^34, 35^. In standard medium, each sperm swam in a random direction, with no clear correlation of orientation among sperm (Fig. 1a; Supplementary Movie 1). When the viscosity of the fluid was increased 36 fold by the addition of 3% PVP, most sperm were still found to swim in uncorrelated directions (Fig. 1b; Supplementary Movie 2). Transient pairings of swimming sperm were seen, but rarely^30^. In viscoelastic 1% LC-PAM solution, sperm spontaneously started to swim in dynamic clusters (Fig. 1c; Supplementary Movie 3). Within each cluster, sperm oriented in the same direction and had similar speeds. The sperm were not physically bound to each other within each cluster; instead, they associated and dissociated from the cluster. Clusters also split or formed dynamically. Even in low cell density, clusters of sperm could still be found, see Supplementary Fig. 2.

We quantified sperm clustering in Fig. 1d. The percentage of sperm that were in a cluster with a size of two or more cells was 21 ± 5% (mean ± s.e.m., *n* = 372) in standard medium, 20 ± 6% in viscous PVP solution (*n* = 278), and 53 ± 5% in viscoelastic LC-PAM solution (*n* = 427). In the case of viscous fluids (no elastic component), most clusters consisted of only two sperm, while in the case of viscoelastic fluid, cluster size varied from 2–16 sperm.

### Sperm orientation correlation, cluster size, and alignment strength increased with fluid viscoelasticity

**Figure 2:**
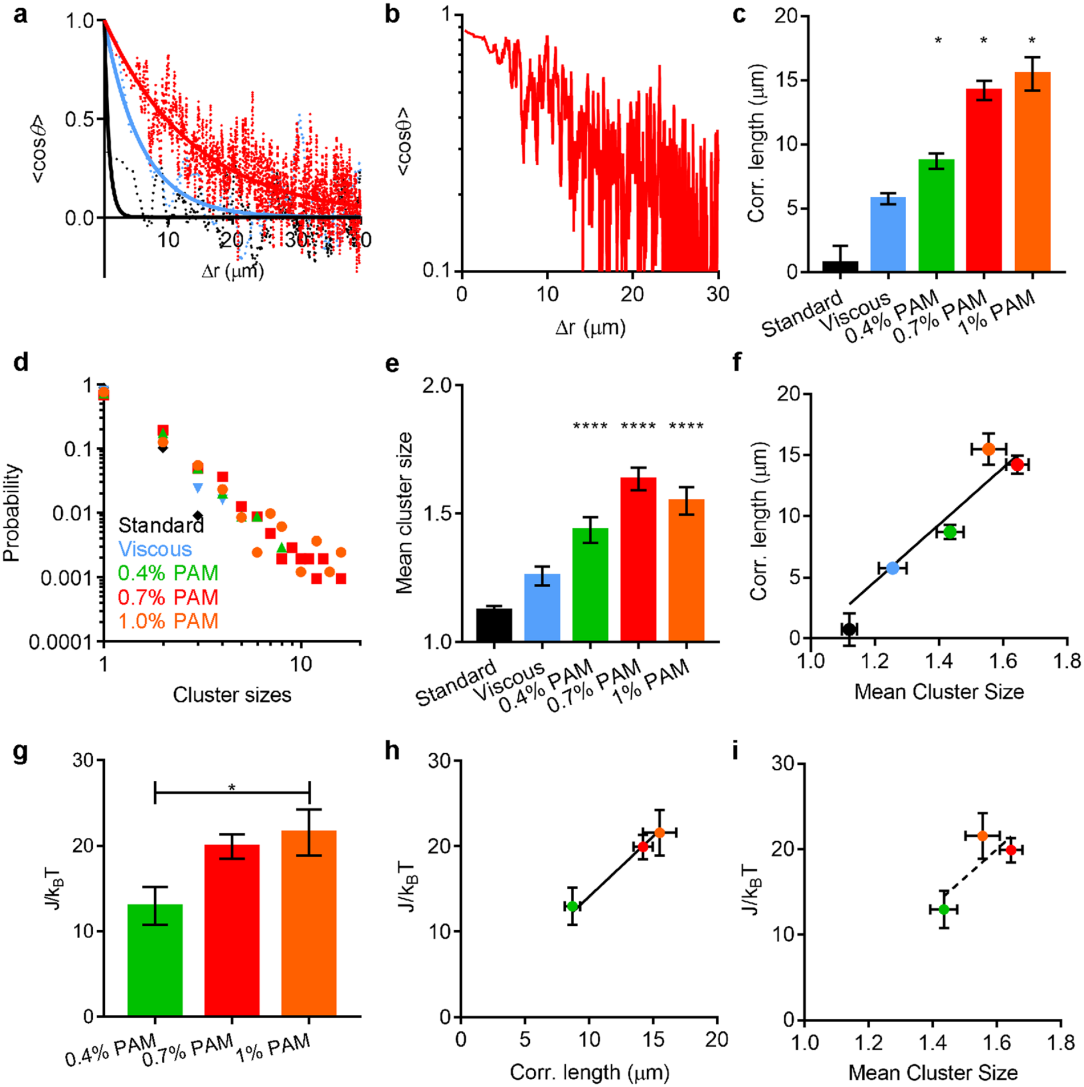
Sperm orientation correlation length and cluster size increase with fluid elasticity. (**a**) The spatial correlation of sperm head orientation as a function of distance in standard medium (black lines), viscous 3% PVP (blue lines) and viscoelastic 0.7% PAM (red lines). Dashed lines are smoothed experimental data and solid lines are fitted data. (**b**) Spatial orientation correlation function exhibits an exponential decay. (**c**) Correlation lengths of sperm in different media. *p < 0.05 from non-overlapping 95% confidence levels, compared with standard medium. (**d**) Distributions of sperm cluster size in different media in log-log scale. 1 = single cell. Cluster size distributions follow a power-law function. (**e**) Mean cluster sizes of sperm in different media. ****p < 0.0001, compared to standard medium, from one-way ANOVA. (**f**) Mean correlation length plotted against mean cluster size. Linear regression shows a line with a slope that is significantly non-zero (p < 0.0001), and intersects the x axis at *x* = 1.0. Data derived from analyses of 5392 cells. Pearson r = 0.97. (**g**) Alignment coupling strength in different concentration of PAM solutions. *p < 0.05 from one-way ANOVA; s.e.m. are plotted, N = 10. (**h**) Coupling strengths and correlation lengths are highly correlated. Spearman r = 1 and p = 0.015. (**i**) Coupling strengths and average cluster sizes are less correlated with Spearman r = 0.81 and p = 0.39.

We computed orientation correlation function for sperm swimming in the three different types of fluids. All orientation correlation functions followed exponential decay curves (Fig. 2a,b). We fitted the experimental correlation function *C*, to e^−*r*/*ξ*^, where *r* is the distance between two sperm and *ξ* is the correlation length. The fitted correlation length is 0.7 ± 1.3 μm (mean ± s.d.) in standard medium, 5.8 ± 0.4 μm in viscous PVP solution, and 15 ± 1 μm in 1% viscoelastic LC-PAM solution (Fig. 2c). Interestingly, when elasticity was introduced into the fluid, the correlation length was increased significantly. When 0.4% of LC-PAM was added to the standard medium, the correlation length was 8.7 ± 0.6 μm, and it further increased to 14.2 ± 0.8 and 15 ± 1 μm in 0.7 and 1% LC-PAM, respectively (Fig. 2c). Note that 0.7% LC-PAM has the viscoelasticity closest to that of bovine cervical mucus (See Supplementary Fig. 1), and was previously used to model the viscoelasticity of bovine cervical mucus during the oestrous (fertile) period of cows^21^.

Since the spatial orientation correlation functions exponentially decayed to 0, there was no sample-wide orientation order in our system. Instead, alignment was a local phenomenon within clusters. This is in agreement with our general observation that orientation alignment only exists within clusters, and there is no orientation correlation between cells outside of clusters. The coexistence of both ordered and disordered phases is a signature of a first-order phase transition, and in such a system the correlation length roughly represents the average size of the ordered phase^7^.

To examine the relationship between cluster sizes and correlation lengths, cluster sizes were independently measured. As shown in Fig. 2d, the cluster sizes were larger in the viscoelastic fluid than in the purely viscous fluid. This effect of viscoelasticity is also reflected in the average sizes shown in Fig. 2e. Average cluster size was found to increase with the viscoelasticity of the fluid (we note that a one-cell cluster means an independently swimming sperm). Interestingly, the distribution of cluster size demonstrates a power law (Fig. 2d). If fitted into

1 $$P(n)=A{n}^{-b},$$

where *n* is the number of cells in each cluster and A is a normalization coefficient, we found that the exponent *b* = 2.9 ± 0.3, 2.7 ± 0.2, 2.4 ± 0.3 (mean ± s.d. from curve fitting) for 0.4, 0.7, and 1% LC-PAM, respectively. For comparison, *b* = 3.0 ± 0.1 in the viscous PVP fluid. This is consistent with the cluster size analysis in swarming or gliding bacteria^1, 36^ and herding animals^37^, with two differences: our sample size did not provide an exponential truncation, and our exponents are larger than their *b* ≤ 2.

Correlation length and average cluster size were generally positively correlated, with a slight exception, since cluster size also depends on cell density, which could not be precisely controlled in the experiment (Fig. 2f). We note that the fitted straight line passes through the point (0,1), which means that the correlation length *ξ* = 0 when the average cluster size is 1. This is consistent with the notion that when all cells swim independently, the correlation length is 0. This linear correlation has also been found in bird flocks^3^.

We further employed a machine learning algorithm based on minimization of Kullback-Leibler divergence to determine the strengths for two adjacent sperm in a cluster to align their directions with each other^38^. Clusters with cells lined up next to each other were chosen and analyzed. We approximated them as a 1D XY model, and used the algorithm to determine the dimensionless alignment coupling strength (*J*/*k*_*B*_ *T*). In 0.4% LC-PAM, the coupling strength was measured as 13 ± 2 (mean ± s.e.m., *n* = 10), and the coupling strength increased with increasing viscoelasticity (Fig. 2g): 20 ± 1 in 0.7% LC-PAM and 22 ± 3 in 1% LC-PAM. As expected, coupling strengths were found to be correlated with the correlation lengths (Fig. 2h), but less correlated with the average cluster sizes (Fig. 2i). This is expected since, while both correlation lengths and cluster sizes are correlated with the interaction strengths, cluster sizes also depend on cell densities, which may vary from time to time^1^.

Both correlation length and coupling strength measure the tendency of nearby particles to align their orientations. They are complementary to each other in a sense that the correlation length is estimated from several fixed time frames, whereas coupling strength was fitted from the statistical average over consecutive time frames throughout a dynamic fluctuating process. Our quantitative analysis in Fig. 2h further shows they are proportional to each other (*p* = 0.015).

### Role of viscoelasticity in sperm-sperm interaction

**Figure 3:**
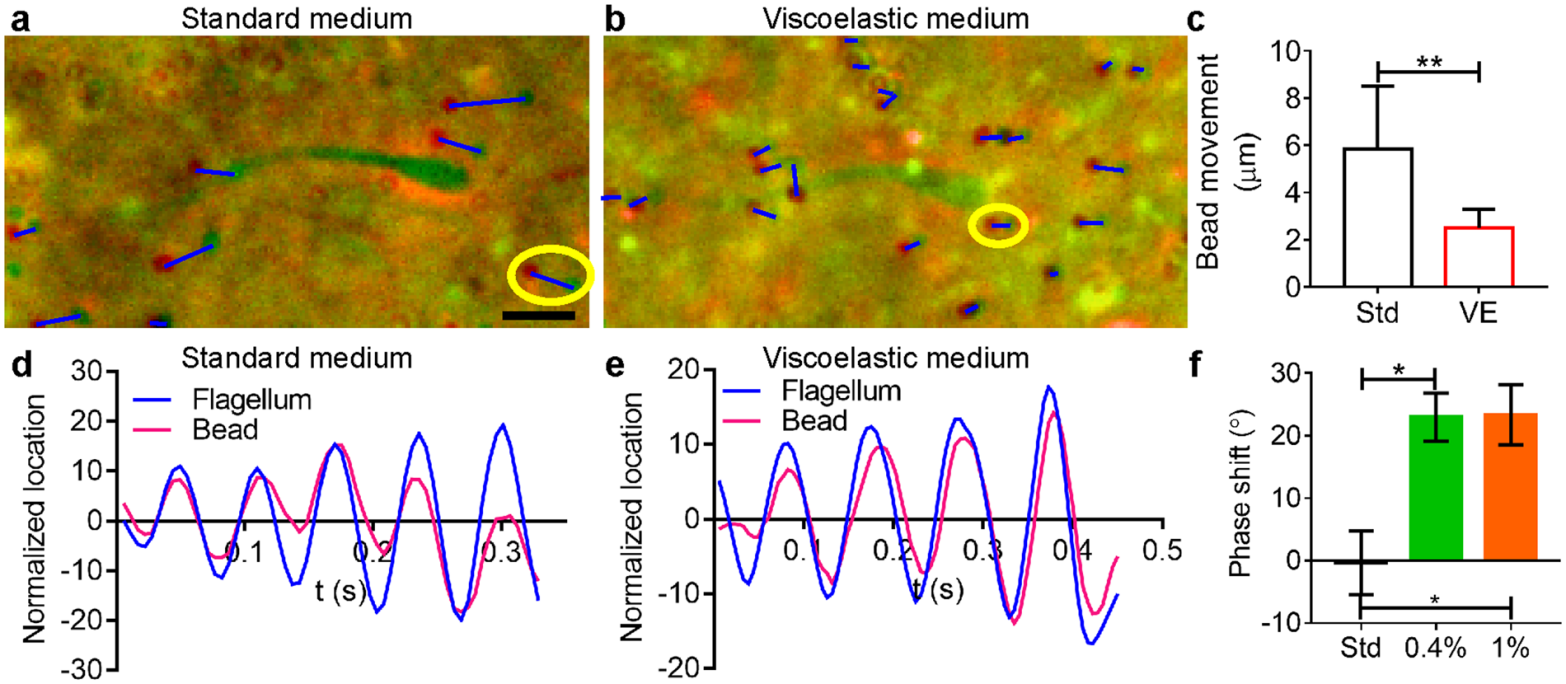
Role of elastic coupling revealed by bead tracking. (**a**,**b**) Bead displacements caused by nearby swimmers, shown by superposition of two consecutive images, the first coloured red and the second coloured green. (**a**) Images of sperm and beads in standard medium had been taken 0.54 sec apart (initial: red, final: green, scale bar: 10 µm). (**b**) Images of sperm and beads in viscoelastic medium (0.7% LC-PAM) had been taken 1.51 s apart. Blue lines were added to show the distance moved by each bead. Yellow circles in (**a**) and (**b**) denote the beads used for phase tracking in (**d**) and (**e**). (**c**) Bead movements are significantly lower in viscoelastic medium than in standard medium (p = 0.0013, Mann-Whitney test, N = 8,20), indicating that sperm generate a smaller flow field in viscoelastic medium. (**d**,**e**) Oscillation of a sperm flagellum and that of the nearest bead. The flagellum oscillation is reported as the location of the point on the flagellum that is directly above the bead. In standard medium (**d**), the oscillation of bead and flagellum are synchronous. In viscoelastic medium (**e**), oscillation of the bead lags behind flagellar movement. (**f**) In standard medium, the average phase difference between the flagellum and the bead is close to 0, while in both viscoelastic media (0.4% and 1.0% LC-PAM) there is a 20° lag, indicating that elasticity plays a role in the mechanical coupling (*p* = 0.02, 0.03, one-way ANOVA, N = 8,8,7).

To understand the role of viscoelasticity of the fluid in sperm-fluid, and thus sperm-sperm interaction, we investigated the fluid movement near sperm using beads as tracers. The fluid movement is measured as bead displacement derived from two consecutive images taken 0.54 s apart and superimposed with color coding (green at t = 0, and red at t = 0.54 s, see Fig. 3a,b). Blue lines were added to connect bead locations at the two time points. It can be seen from the images that the net bead movement was higher in standard medium than in viscoelastic media, meaning sperm generated larger flow fields in standard medium than in viscoelastic media. The quantification of the bead movements is shown in Fig. 3c.

In low Reynolds number incompressible Newtonian fluid, if the flow is driven by a prescribed motion of a boundary, the fluid velocity at each point must be linear in the rate of motion of the boundary at the same time (quasi-steady). In our case, the beating flagellum provided the moving boundary; therefore, bead movement and flagellar beating should have been, and was, essentially in phase in the Newtonian fluids (Fig. 3d). On the other hand, when the material between the moving boundary and the bead was elastic, the material was compressed when the boundary moved toward the bead, and relaxed shortly after the boundary reversed direction. This deformation resulted in a phase lag between the movements of the beating flagellum and the bead, as shown in Fig. 3e. On average, we observed 0 ± 5° (mean ± s.e.m., *n* = 8) phase difference in standard medium vs. 23 ± 4° (*n* = 8) and 23 ± 5° (*n* = 7) for 0.4% and 1% LC-PAM, respectively (Fig. 3f).

The phase lag observed here further demonstrates the role of viscoelasticity in sperm - fluid interaction, pointing to a possible sperm – sperm interaction mechanism in which sperm are coupled by the elastic component of the fluid. This is consistent with previous theoretical calculations. In Li *et al*.^39^, it was numerically shown that the polymer stress enhances the local clustering and polar alignment of pusher swimmers^39, 40^. In Ferrante *et al*., it was shown that collective movement of microswimmers can emerge through energy cascading to the zero-phonon ground state in a purely elastically coupled system^41^. Other possible causes for the collective swimming are considered and rejected in the Supplementary Discussion.

### Single sperm swimming behavior was modulated by viscosity and viscoelasticity

**Figure 4:**
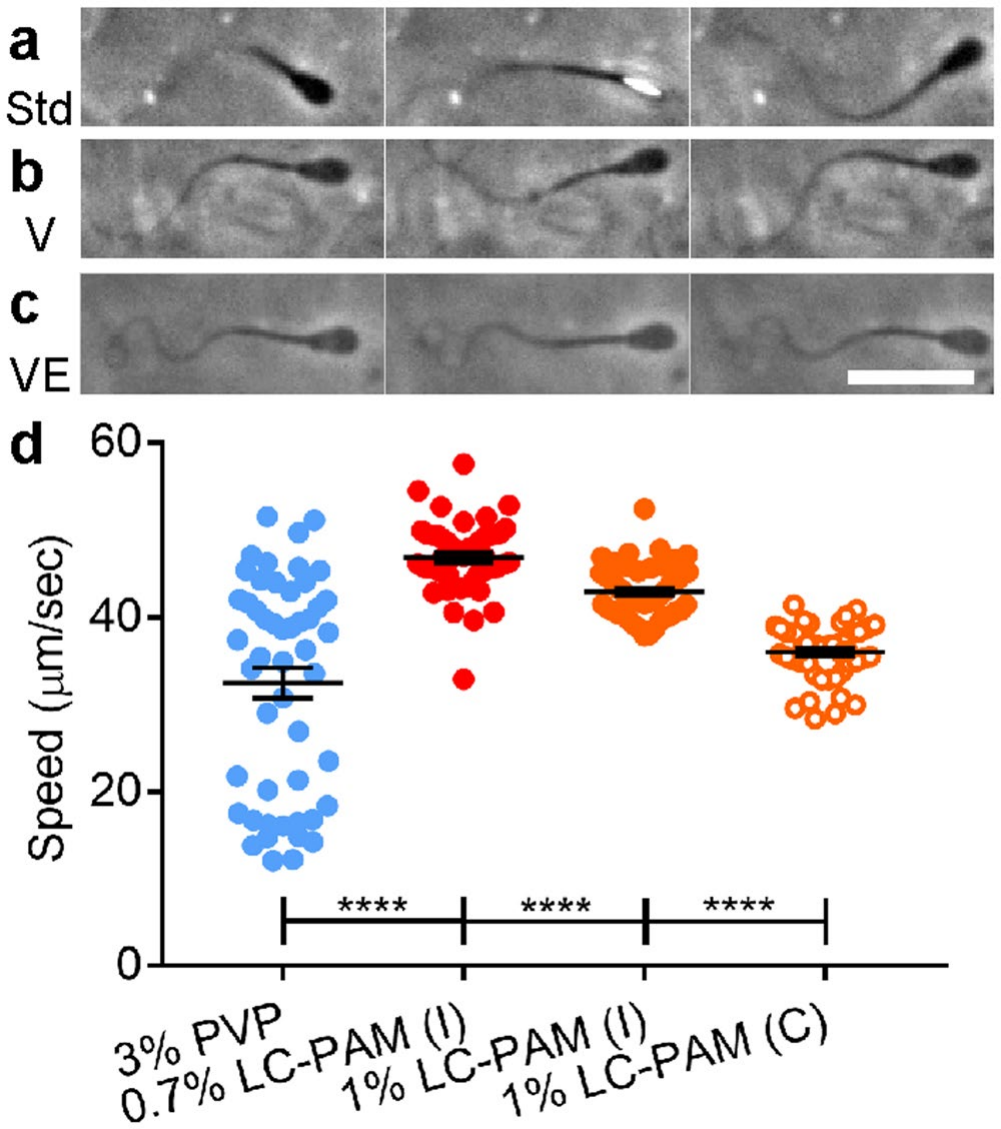
Influence of fluid rheology on sperm motility. (**a**) In standard medium, sperm exhibit near-surface swimming and rolling of the whole body, evidenced by alternating broad (black) and narrow (bright) surfaces of the head. (**b**) In a Newtonian viscous fluid (3% PVP), more than half of the sperm swam without rolling, and showed two-dimensional beating of the flagellum. Scale bar: 25 μm. (**c**) In viscoelastic medium (1% LC-PAM), most sperm swam without rolling by using two-dimensional beating of the flagellum. (**d**) While 3% PVP is less viscous than the solutions of LC-PAM, sperm swam slower in it, indicating viscoelasticity allows sperm to swim faster. When we compared single sperm (1% PAM, orange filled circles) against those in clusters (orange open circles), it was found that sperm swimming in clusters had a lower mean speed. This is compared with 133 ± 2 µm/s (mean ± s.e.m.) of single sperm in standard medium (****p < 0.0001, Mann-Whitney test, N = 50). Deborah numbers of individually swimming sperm in 0.7 and 1% LC-PAM are estimated to be 0.51 and 2.0 respectively.

In standard medium, single sperm swam via a rolling motility mode, where head rotation could be clearly seen (Fig 4a and Supplementary Movie 4)^30, 31, 42^. In contrast, in Newtonian viscous medium (3% PVP), > 65% of the sperm swam via a slithering motility mode in which the flagellum beat in a single plane parallel and very close to a surface (Fig. 4b and Supplementary Movie 5). In viscoelastic medium (0.4–1% LC-PAM), 75% or more of the sperm exhibited slithering (Fig. 4c and Supplementary Movie 6)^21, 30^. These observations are consistent with previous reports^21, 30^. It is interesting to note that sperm switched from rolling to slithering when the viscosity increased^30^. Nevertheless, collective dynamics occurred only in viscoelastic fluid, suggesting that the change to slithering movement was not sufficient to cause the collective dynamics.

Interestingly, we also found that sperm swam faster in viscoelastic fluids than in Newtonian viscous fluids of equivalent or lower viscosity (Fig. 4d). The average sperm speed is 32 ± 2 µm/s in viscous fluid (3% PVP), in contrast to 46.8 ± 0.6 µm/s in viscoelastic fluid with Deborah number 0.51 (0.7% LC-PAM). This is consistent with reported theoretical prediction^43–45^. Since sperm collective and independent swimming can be found at the same time in viscoelastic fluid, we next compared the speeds of collective and independent swimming sperm within the same fluid. Figure 4d shows that sperm swimming collectively moved slower than sperm swimming individually. There are mouse species in which the sperm join together to form cooperative groups; the joined sperm swim faster than individually-moving sperm^26, 27, 46^. However, this relationship did not apply to the collective movement we observed. We should note that the collective behaviour we observed here is different from that of the previously-reported cooperative groups of attached sperm, because the sperm in our experiments moved in and out of groups dynamically rather than remaining together in the clusters.

## Discussion

We report a collective swimming pattern of sperm enabled by the elastic component of complex fluids. This result highlights the significance of viscoelastic properties of fluid in assisting cell – cell interaction, and as such cells are mechanically inter-linked through the viscoelastic fluids to form organized swimming patterns. Our results raise the questions of whether biological fluid evolved viscoelasticity in order to facilitate cell-cell communication, and whether the collective sperm swimming induced by viscoelasticity provided an evolutionary pathway for the development of the cooperative attachments known to occur between sperm in some rodent species^26, 27^. Previous work on the effects of fluid properties on sperm swimming behaviour has largely focused on viscosity^30, 47^. Nevertheless, it is known that most bodily fluids, including cervical mucus, are viscoelastic; therefore, it is important to explore whether/how viscoelasticity of fluids play a role in sperm swimming behaviour^20, 21, 48^. We note that our experimental work is consistent with recent numerical computations, in which pusher swimmers were predicted to swim collectively in viscoelastic fluids^39, 41^. Further experiments are required to determine whether this collective swimming improves the efficiency of sperm movement through the female tract.

Further, our system demonstrates a good model for studying collective dynamics of active matter systems that consist of living cells. In recent years, a number of artificial systems have been developed to determine how well-defined particle-particle interactions at micro-scale lead to collective motion at macro-scale^49–51^. When spherical colloidal particles propel themselves through chemical reactions and interact with each other by osmotic and phoretic effects, they phase separate into a dense, collective solid phase and a loose, disordered gas phase^50^. However, phase coexistence has not been reported in living systems thus far^52, 53^. The observation of the collective swimming here is largely in agreement with other biological collective movements^1, 37^. So far, collective dynamics in biology has mostly been analysed phenomenologically in bird flocking^3, 4^, and our results here provide a different inroad to the general phenomenon of biological collective movement. Since the flocking transition was found to closely resemble liquid-gas phase transition^54^, and the sperm collective swimming observed here is an example of the flocking transition, our system can provide insights into linking the general phenomenon of liquid-gas transition to collective dynamics in biology.

## Methods

### Reagents and media

Unless otherwise noted, chemicals were purchased from Sigma–Aldrich. Tyrode albumin lactate pyruvate (TALP, referred to here as standard medium) medium^55^ consisted of 99 mM NaCl, 3.1 mM KCl, 25 mM NaHCO_3_, 0.39 mM NaH_2_PO_4_, 10 mM HEPES-free acid, 2 mM CaCl_2_, 1.1 mM MgCl_2_, 25.4 mM sodium lactate, 0.11 mg/mL sodium pyruvate, 5 mg/mL gentamicin, and 6 mg/mL BSA (Fraction V; Calbiochem, La Jolla, CA, USA), titrated with 1 M HCl to a pH of 7.4 and 300 mOsm/kg. Polyvinylpyrrolidone (PVP, 360 kDa) and long-chain or short-chain polyacrylamide (LC-PAM, 5–6 MDa or SC-PAM, 400 kDa) were added to the standard medium to make the Newtonian viscous and viscoelastic fluids, respectively. All fluids were equilibrated in a 38.5 °C incubator (bovine body temperature) with 5% CO_2_ in humidified air before use.

### Bovine sperm preparation

Fresh bull semen samples were generously provided by Genex Cooperative, Inc. (Ithaca, NY, US). Shortly after semen collection, 1 mL of bovine semen sample was diluted in 5 mL of warm standard medium. The tubes were kept in 38.5 °C water bath (bovine body temperature), and then transported to the lab in a warm water jacket. Semen samples arrived at the lab within 1 hr of collection. The semen samples were then centrifuged at 200 × g for 5 min. After the removal of the supernatant, 5 mL of standard medium were added to resuspend the sperm pellet, and then another 5 min of 200 × g centrifugation followed. The pellet was resuspended with 1 mL of standard medium (120–150 million cells/mL). Depending on the cell concentration, the suspension was further diluted in medium (typically with a 2:5 dilution). Experiments were performed independently with semen from two to three bulls for each condition. Collective dynamics in LC-PAM was observed in all (n = 7) bulls.

### Microfluidic assays

Detailed microfluidic device design can be found in Tung *et al*.^31^. Devices were made of polydimethylsiloxane (PDMS), using standard stamping techniques, with an etched silicon wafer treated with (1 H,1 H,2 H,2H-perfluorooctyl) trichlorosilane (FOTS). The viewing chamber was 2.47 mm wide, 2 cm long, and 120 µm deep. The glass slide was coated with PDMS before bonding with the PDMS device using oxygen plasma. Prior to the experiments, channels were filled with standard medium (to be used as control) or corresponding polymer dissolved in standard medium and equilibrated overnight with 5% CO_2_ in humidified air at 38.5 °C (bovine core body temperature). For the experiments, the microfluidic devices were placed on a temperature controlled glass plate (OkoLab) on a heated microscope stage (Carl Zeiss), which were kept at 38.5 °C. Sperm were seeded into one end of the device to allow them to swim into the chamber.

### Cell imaging and analysis

Images of sperm swimming on a flat PDMS surface were acquired either at 17.8–19.55 or 180–190 FPS using phase contrast microscopy (10X objective) by a Neo sCMOS high-speed digital camera (Andor) and the NIS Elements 4.0 imaging software (Nikon). To analyse the sperm movement in the digital video recordings, the locations of sperm heads and the orientation of the sperm were tracked manually from image to image using ImageJ and MATLAB. For speed analysis, the centroid of each sperm head location was tracked using Manual Tracking plugin in ImageJ from 17.8–19.55 FPS videos at the same rate as the video frame rate. The average speed of one track was calculated as the sum of displacement/time. Detailed method can be found in Tung *et al*.^42^.

### Orientation, cluster, and correlation analyses

In each image being analysed, the orientations ($\hat{s}$) and head locations ($\mathop{r}\limits^{\rightharpoonup }$) of all sperm were tracked manually using ImageJ. For cluster analysis, a MATLAB program was used to analyse the location and orientation data, so that two sperm were considered to be within the same cluster when their head separation was within 17.5 μm and the orientation difference was within 20°. General trend of cluster sizes is not sensitive to this definition, see Supplementary Fig. 3. The orientation correlation function is defined as

2 $$C(r)={\langle ({\hat{s}}_{i}\cdot {\hat{s}}_{j})\delta ({\mathop{r}\limits^{\rightharpoonup }}_{i}-{\mathop{r}\limits^{\rightharpoonup }}_{j}-\mathop{r}\limits^{\rightharpoonup })\rangle }_{ij}={\langle \cos {\theta }_{ij}\delta ({\mathop{r}\limits^{\rightharpoonup }}_{i}-{\mathop{r}\limits^{\rightharpoonup }}_{j}-\mathop{r}\limits^{\rightharpoonup })\rangle }_{ij},$$

where *i* and *j* label the sperm, $\mathop{r}\limits^{\rightharpoonup }$ is the distance between the two cells, ${\theta }_{ij}$ is the angle between ${\hat{s}}_{i}$ and ${\hat{s}}_{j}$, and ${\langle \ldots \rangle }_{ij}$ represents the average over all possible pairs. The correlation values were then fitted into $C={e}^{-r/\xi }$, where *ξ* provides the correlation length.

### Estimating the 1D XY model coupling strength (*J*/*k*_B_*T*)

We observed that there were many one-dimensional clusters (cells lined up next to each other) composed of *N* particles (*N* = 4–8), and remained together for several seconds. The stability of these clusters implies that the cells within the same cluster tended to align their moving directions (therefore the orientations) with one another. We used 1D XY model to describe the velocity coupling between adjacent particles:

3 $$H(\{\hat{s}\})=-J\sum _{i=1}^{N-1}{\hat{s}}_{i}\cdot {\hat{s}}_{i+1}.$$

Here ${\hat{s}}_{i}$ represents the orientation of the $i$ ^th^ cell, and the coupling constant $J$ has the dimension of energy, and quantifies how strongly two adjacent cells align their orientations. A larger $J$ corresponds to a stronger tendency for two adjacent particles to have the same orientation. Considering the effect of randomization, a dimensionless $J/{k}_{B}T$ was used to describe overall alignment strength. Statistically, the model implies that the probability of one orientation distribution $\{\hat{s}\}=({\hat{s}}_{1},\,{\hat{s}}_{2},\ldots ,{\hat{s}}_{N})$ is proportional to ${e}^{-H(\{v\})}$, or follows the Boltzmann distribution. To fit the coupling $J/{k}_{B}T$ from the data, we tracked the clusters for 2–3 sec (41–62 frames), and recorded the orientation distributions. We then minimized the Kullback-Leibler divergence, using the method developed in Lin *et al*.^38^ to obtain the coupling strength $J/{k}_{B}T$.

### Rheology measurements

Rheology of the polymer (LC-PAM, SC-PAM, and PVP) solutions were measured using a rotational shear rheometer (TA Instruments, DHR3) with a standard cup and DIN rotor at 38.5 °C. Viscosity was measured using continuous flow mode, and the storage modulus was measured using oscillation mode with 0.1% maximum strain. Viscosity of the PVP solutions were derived using Fikentscher K value. Cervical mucus samples from oestrous cows had been measured using the same rheometer with 25 mm stainless steel parallell plates^21^.

## Electronic supplementary material

Supplementary Info

Supplementary Movie 1

Supplementary Movie 2

Supplementary Movie 3

Supplementary Movie 4

Supplementary Movie 5

Supplementary Movie 6
