# Supplementary material for: Fluid viscoelasticity promotes collective swimming of sperm
Source: Sci Rep. 2017 Jun 9;7:3152. doi: 10.1038/s41598-017-03341-4 (PMC5466690; doi:10.1038/s41598-017-03341-4)
Supplement: Supplementary file 1 — Supplementary Info [file 41598_2017_3341_MOESM1_ESM.pdf]

Supplementary Information

for

Fluid viscoelasticity promotes collective swimming of sperm

Authors: Chih-kuan Tung[1,2,3,*], Chungwei Lin[4], Benedict Harvey[1], Alyssa G. Fiore[2], Florencia Ardon[1], Mingming Wu[2,*], and Susan S. Suarez[1,*]

[1]Department of Biomedical Sciences, Cornell University, Ithaca, NY, 14853; [2]Department of Biological and Environmental Engineering, Cornell University, Ithaca, NY 14853; [3]Department of Physics, North Carolina A&T State University, Greensboro, NC 27411; [4]Mitsubishi Electric Research Laboratories, Boston, MA 02139

*Correspondence should be addressed to C.K.T. (email: ctung@ncat.edu), M.W. (email: mw272@cornell.edu), and S.S.S. (email: sss7@cornell.edu).

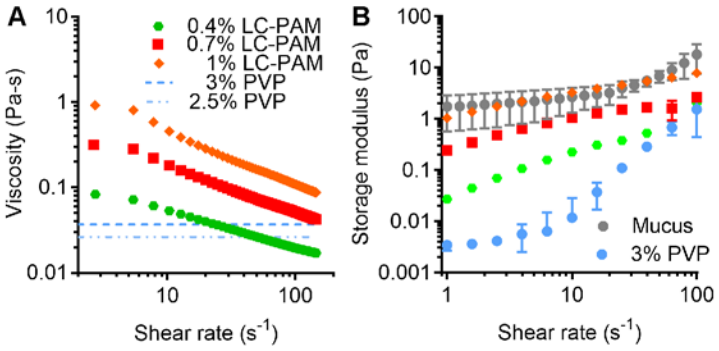

Supplementary Figure 1. Viscosity and elasticity measurements of the polymer solutions. (**a**) Viscosity of different media. Newtonian viscous medium (2.5% and 3% PVP), has a near constant viscosity (formula viscosity shown), while viscoelastic medium (LC-PAM) is shear-thinning with a power-law decay. Both 0.7 and 1% LC-PAM solutions are more viscous than 3% PVP at all measured shear rates, while 0.4% LC-PAM is comparable with the PVP solutions at certain shear rates. (**b**) Elasticity (represented by storage modulus) of different media compared with estrous bovine cervical mucus. The 0.7% LC-PAM solution, which was used to model bovine cervical mucus, has comparable moduli as the mucus samples, while 3% PVP solutions are less elastic than any of the mucus samples.

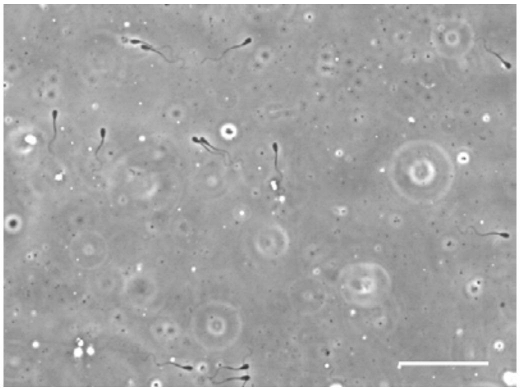

Supplementary Figure 2. Sperm forming clusters (pairs, cluster size = 2) in low cell density limit.

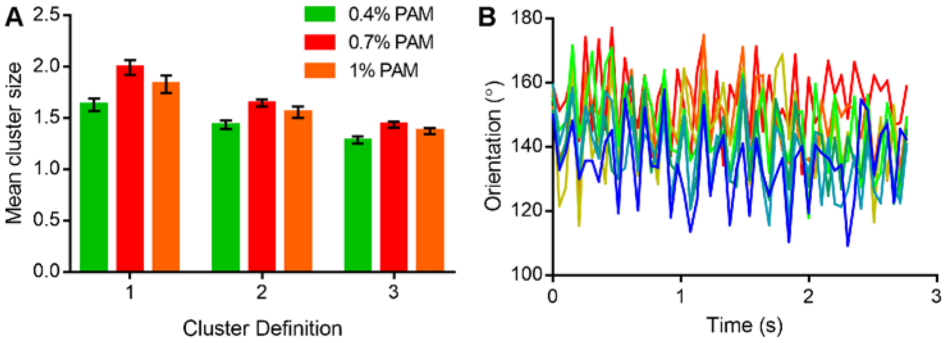

Supplementary Figure 3. Definition of clusters does not influence the general trend of the observation. (**a**) Average cluster sizes according to three different definitions: (1) within 12.25 mm separation and 20° angular difference; (2) 17.25 mm separation and 20° angular difference, used in main text; (3) 22.25 mm

separation and 25° angular difference. While the definition changes the exact value, the trend between samples remained the same. (**b**) Angular fluctuation of 7 sperm within a cluster. It can be seen that the orientation of sperm easily fluctuates near 20°.

Supplementary Discussion: **Elimination of other possible mechanisms of collective swimming.**

A number of factors other than the elasticity of the medium could have contributed to the clustering observed, and we examine them here.

(1) Motility induced clustering. In a well-studied system, it was established that spherical colloids form clusters when chemical reactions cause them to propel themselves (1). Their movements result in coexistence between a solid phase (clustered with lattice structure) and a gas phase (dispersed). Clusters formed through collisions, and it was demonstrated numerically that swimming pressure due to collisions caused by the propulsion alone result in clustering, even in the absence of other types of attraction between the self-propelled objects (2, 3). In our experiments, if collision-induced swimming pressure were the dominant mechanism, we should have observed a high degree of sperm clustering in the viscous fluids as well as in the viscoelastic fluids, since collision probability enhancement would occur in both cases due to increased slithering of sperm The slight increase in correlation length in viscous solutions in contrast to that in the standard medium is likely to have contributed to increased slithering motility in the PVP solution. In standard medium, where rolling motion dominates, the sperm head is on average 5 μm from surfaces (4), while in viscous PVP solution, it is less than 1 μm from surfaces (5). This is because slithering motion keeps the head much closer to a surface. As a result, slithering sperm are likely to collide more often with other sperm than sperm in standard medium (Figure 1A,B; and Video 1, 2).

(2) Shear thinning promotes aggregation of settling spheres. It has been proposed that the shear-thinning property of viscoelastic fluids can create a corridor of fluid with lower viscosity for two settling spheres to aggregate together, since the settling spheres shear the fluid and therefore lower the viscosity (6). In that scenario, viscoelasticity allows settling spheres to generate larger flow fields. Because we showed that the flow field generated by sperm in viscoelastic fluids was actually smaller than that in the standard medium, where no collective swimming was observed, this shear-thinning flow field enhancement is unlikely to be the dominant mechanism for the emergence of collective swimming.

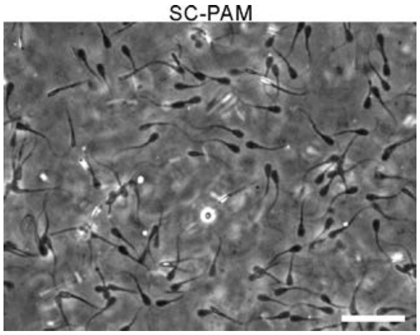

Supplementary Figure 4. No clustering was observed in SC-PAM solution with the same weight percentage (0.7%) as LC-PAM. Scale bar: 50 µm.

(3) Chemically induced sperm collective swimming. Here we consider two plausible chemical effects, chemical repulsion and flocculation. If PAM molecules and sperm surfaces repel each other due to chemical components (such as charges), it would be energetically more favourable for the sperm heads to be in touch with each other. If the functional groups of PAM react with sperm surfaces to form chemical bonds, PAM could form bridges that link sperm together (flocculation). We tested these two scenarios by comparing sperm swimming patterns in solutions of short-chain PAM (400 kDa, SC-PAM) and LC-PAM. At the same weight percentage (0.7%), SC-PAM and LC-PAM solutions have the same concentrations of functional groups that could be repelling or linking sperm, yet with different

rheological properties. Due to its reduced chain length, SC-PAM is both less viscous and less elastic than LC-PAM. In Supplementary Figure 4, it can be clearly seen that the orientations of sperm in SC-PAM are random and no collective swimming can be seen, just like sperm in standard medium. This eliminates the possibility that chemical effects between PAM and sperm surfaces is the cause of clustering. Note that, while SC-PAM is much smaller than LC-PAM, the molecules are still large and sufficient in size to cause flocculation. For example, 120 kDa branched PAM has been used for flocculation in water treatment (7).

(4) Collective swimming due to depletion interaction. Depletion interaction is an entropic force that results from the non-negligible sizes of the depletion molecules (polymers). Given that the radius of gyration scales as a square root of the polymer length, the radii of gyration of the PVP and SC-PAM are not orders of magnitude smaller than the LC-PAM, therefore offering relatively similar (at least similar orders of magnitude) exclusion zones (8). Because the difference in depletion interaction is not as drastically different between LC-PAM and PVP/SC-PAM, yet the amount of clustering is dramatically lower in PVP/SC-PAM, it is unlikely that the collective swimming is caused by depletion interaction. Furthermore, we know that bovine sperm surfaces have a net negative charge (9), while the amine groups make PAM less negatively charged than PVP, making PAM less likely to be depleted from the zone close to sperm surface than PVP. Since the two polymers carry similar charges otherwise, depletion should be a stronger effect in PVP than in PAM; therefore, depletion is not a major contributor to the clustering of sperm.

1. Palacci J, Sacanna S, Steinberg AP, Pine DJ, Chaikin PM. Living Crystals of Light-Activated Colloidal Surfers. Science. 2013;339(6122):936-40.

2. Fily Y, Marchetti MC. Athermal Phase Separation of Self-Propelled Particles with No Alignment. Phys Rev Lett. 2012;108(23):235702.
3. Takatori SC, Yan W, Brady JF. Swim Pressure: Stress Generation in Active Matter. Phys Rev Lett. 2014;113(2):028103.
4. Tung C-K, Ardon F, Roy A, Koch DL, Suarez SS, Wu M. Emergence of Upstream Swimming via a Hydrodynamic Transition. Phys Rev Lett. 2015;114(10):108102.
5. Nosrati R, Driouchi A, Yip CM, Sinton D. Two-dimensional slither swimming of sperm within a micrometre of a surface. Nat Commun. 2015;6:8703.
6. Joseph DD, Liu YJ, Poletto M, Feng J. Aggregation and dispersion of spheres falling in viscoelastic liquids. J Non-Newtonian Fluid Mech. 1994;54:45-86.
7. Nagy DE, Williams LL, Coscia AT, inventors; American Cyanamid Co, assignee. Process of manufacturing polyacrylamide-based flocculant and flocculations therewith. US Patent 3488720 A1970 Jan 6, 1970.
8. Lekkerkerker HNW, Tuinier R. Colloids and the Depletion Interaction: Springer Netherlands; 2011. 234 p.
9. Iqbal N, Hunter AG. Comparison of Various Bovine Sperm Capacitation Systems for Their Ability to Alter the Net Negative Surface Charge of Spermatozoa. J Dairy Sci.78(1):84-90.

Supplementary Movie 1. Sperm swimming in standard medium. No collective swimming was observed.

Supplementary Movie 2. Sperm swimming in viscous PVP solution. No collective swimming was observed.

Supplementary Movie 3. Sperm swimming in viscoelastic PAM solution. Sperm formed dynamic clusters.

Supplementary Movie 4. Sperm rolling motility in standard medium.

Supplementary Movie 5. Sperm slithering motility in viscous PVP solution.

Supplementary Movie 6. Sperm slithering motility in viscoelastic LC-PAM solution.
